# Low Serum Uric Acid Predicts Risk of a Composite Disease Endpoint

**DOI:** 10.3390/medicina57040361

**Published:** 2021-04-08

**Authors:** Fatma Özpamuk-Karadeniz, Yusuf Karadeniz, Adnan Kaya, Servet Altay, Günay Can, Altan Onat

**Affiliations:** 1 Departments of Cardiology, Special Büyükşehir Hospital, 42010 Konya, Turkey; 2 Division of Endocrinology and Metabolism, Department of Internal Medicine, Faculty of Medicine, Necmettin Erbakan University, 42010 Konya, Turkey; dryusufkaradeniz@gmail.com; 3 Department of Cardiology, Special Memorial Hospital Bahçelievler, 34100 Istanbul, Turkey; adnankaya@ymail.com; 4 Department of Cardiology, Faculty of Medicine, Trakya University, 22020 Edirne, Turkey; svtaltay@gmail.com; 5 Departments of Public Health, Cerrahpaşa Medical Faculty, Istanbul University-Cerrahpaşa, 34098 Istanbul, Turkey; gunaycan09@yahoo.fr; 6 Departments of Cardiology, Cerrahpaşa Medical Faculty, Istanbul University-Cerrahpaşa, 34098 Istanbul, Turkey; alt_onat@yahoo.com.tr

**Keywords:** serum uric acid, coronary heart disease, diabetic status, mortality, smoking status, total cholesterol

## Abstract

*Background and objectives:* Mortality may increase in hypouricemia as well as inhyperuricemia. We assessed the predictive value of low serum uric acid (SUA) levels on the risk of overall mortality or a composite endpoint of death and nonfatal events. *Materials and Methods:* In 1013 community-based middle-aged adults, free of uncontrolled diabetes and coronary heart disease at baseline, the association of sex-specific SUA tertiles with defined outcomes was evaluated prospectively by logistic regression, stratified to gender and presence of type-2 diabetes, using recent criteria. *Results*: Totally, 43 deaths and additional incident nonfatal events in 157 cases were recorded at a median 3.4 years’ follow-up. Multivariable linear regression disclosed SUA to be significantly associated among non-diabetic individuals positively with creatinine, triglycerides, and body mass index in women further with fasted glucose. In multivariable-adjusted logistic regression analysis, sex-specifically dichotomized baseline uric acid (<5.1 and <4.1 mg/dL vs. higher values) significantly predicted the non-fatal events in the whole sample (relative risk (RR) 1.51 [95% confidence interval (CI) 1.02; 2.26]), as well as in men, while composite endpoint in the whole sample tended to rise (RR 1.38). Compared with the intermediate one, the top and bottom SUA tertiles combined tended to confer mortality risk (RR 2.40 [95% CI 0.89; 6.51]). Adverse outcomes in diabetic women were predicted by tertiles 2 and 3. *Conclusions*: Inverse association of SUA with adverse outcomes, especially in men, is consistent with the involvement of uric acid mass in autoimmune activation. The positive association of uric acid with adverse outcomes in diabetic women is likely mediated by concomitant high-density lipoprotein dysfunction.

## 1. Introduction

Serum uric acid (SUA) is a known endogenous antioxidant, probably functioning through the free radical scavenging capacity [1]. An increase in the SUA level has been considered to represent counteracted oxidative damage in subjects with atherosclerosis, based on the observation that individuals who had carotid intima-media thickness detected had higher serum total antioxidant capacity than matching controls with low thickness [2]. Yet, SUA has complex chemical and biological effects, whereby its pro-oxidant properties may explain the association of its elevated levels with hypertension, metabolic, and vascular diseases [3]. Uric acid is pro-inflammatory in rat vascular smooth muscle cells and stimulates human mononuclear cells to produce cytokines [4]. Observational studies disagree with the relationship of uric acid with mortality, and with factors modifying this relationship [5].

Diabetes was not an independent predictor of hyperuricemia in the Novarra Study [6] and, among diabetic patients, a higher SUA level is not independently associated with the extent of coronary artery disease. A prospective study on 1268 diabetic patients with a 10-year follow-up reported no association between SUA and all-cause or cardiovascular mortality [7].

In contrast, reports evaluating uric acid categories for outcome indicated a disparity in low uric acid levels. A large prospective study in Taiwan reported that sex- and age-adjusted hazard ratios of uric acid levels for overall mortality were increased, not only for hyperuricemia, but also for uricemia lower than 5 mg/dL [8]. This was partly supported in the Scottish Heart Health Extended Cohort Study, wherein the lowest fifth of SUA (<3.7 mg/dL), exhibiting higher prevalence of diabetes and low total cholesterol levels, increases higher cardiovascular (and all-cause) mortality, compared with the second fifth [5].

The controversial nature of the mediation of SUA levels for risk of cardio-renal and metabolic diseases and mortality likely resides in a non-linear risk relationship arising from compounding by theinteraction of several factors interacting, such as sex, presence of glucose intolerance, diabetes mellitus, or coronary heart disease (CHD). Such compounding may be more pronounced in population segments prone to impaired function of high-density lipoprotein (HDL) or other protective plasma proteins and the associated autoimmune activation [9].

Therefore, in this longitudinal study over an intermediate follow-up period evaluating Turkish adults, known to be prone to metabolic syndrome (MetS) and diabetes [10], and attending a major tertiary hospital, we pursued the following objective: the predictive value of SUA concentrations for all-cause mortality, non-fatal cardiac and non-cardiac events, and for a composite endpoint of mortality plus non-fatal events. To better elucidatethe associations, we analyzed the issue by stratifying the patients according to sex and diabetic status.

## 2. Materials and Methods

### 2.1. Study Sample

**Figure 1:**
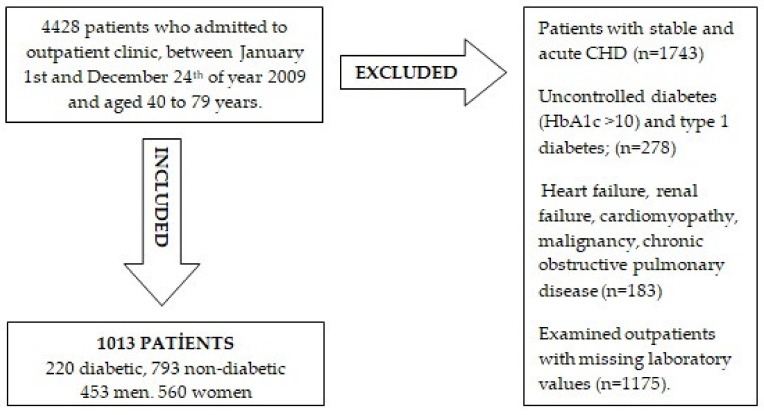
Flow diagram of the study (CHD: chronic heart disease).

The baseline study sample is formed by 1013 outpatients and admitted patients, aged 40 to 79 years, who have applied between 1 January and 24 December of the year 2009 to the SiyamiErsek Center for Cardiovascular Surgery (a large center for individuals suspected of cardiac disease) and had available values for SUA concentrations. A flow diagram with exclusion criteria is summarized in Figure 1. Patients using drugs that lower serum uric acid levels, such as allopurinol, were not included in the study.Follow-up consisted of a median of 3.4 (up to 5.5) years. The study protocol was in accordance with the Declaration of Helsinki and approved by the local Ethics Committee (SEEPK 13.08.2013 date and 2. number decision). Data recorded in the hospital charts were obtained.

### 2.2. Measurement of Risk Factors

Blood pressure (BP) was measured with an aneroid sphygmomanometer (Erka, Germany) in the sitting position on the right arm, and the mean of at least two recordings 5 min apart of registered. BP values were reported in milimeters of mercury (mmHg). Body mass index (BMI) was computed from values of weight/height squared and universally expressed in kg/m^2^, resulting from mass in kilograms and height in meters. The patients were classified as never, former, and current smokers based on their history of cigarette smoking.

### 2.3. Blood Samples

Blood samples were analyzed in a central laboratory after an overnight fast, using Siemens Healthcare Diagnostic Products kits and calibrators (Marburg, Germany). SUA concentrations were measured enzymatically with the uricase method and creatinine, with the Jaffe method having a lower detection limit of 0.1 mg/dL. Serum concentrations of total cholesterol, triglycerides, glucose, and total bilirubin were measured enzymatically. Low-density lipoprotein (LDL)- and HDL-cholesterol were quantified directly with the elimination catalase method and Adviaautoanalyzer. Concentrations of HbA1c were measured in whole blood agglutination inhibition and serum C-reactive protein with latex-enhanced immunoturbidimetry. Left ventricular ejection fraction was calculated using the modified Simpson’s method.

### 2.4. Definitions

Type-2 diabetes mellitus was diagnosed with the criteria of the American Diabetes Association (ADA) [11]. CHD was diagnosed by a history of myocardial infarction (MI) or revascularization, or by greater than 50% stenosis of at least one major coronary artery on angiography.

### 2.5. Outcomes

All-cause mortality was taken as the primary outcome and a composite endpoint as the secondary outcome. The composite endpoint was defined as overall deaths plus incident nonfatal events of CHD, MI, stroke, heart failure, thromboembolism, chronic obstructive pulmonary disease (COPD), and malignancy. Information on the cause of death was drawn from hospital records during follow-up, from records of the nation-wide identity participation system, or first-degree relatives. Chronic CHD denotes patients with coronary disease in whom slow coronary flow or greater than 50% stenosis was identified at the coronary angiogram; included were individuals subjected to percutaneous coronary intervention or coronary artery bypass surgery during the follow-up period. Patients (*n* = 95) giving a history of percutaneous coronary intervention or coronary bypass grafting or MI, all in the follow-up years 2010 through 2014, were also defined as such. Congestive heart failure was diagnosed when typical symptoms (e.g., breathlessness, ankle swelling, and fatigue) and signs (e.g., elevated jugular venous pressure, pulmonary crackles, and displaced apex beat) resulted from an abnormality of cardiac structure. Stroke was defined as an impairment of brain function that resulted in an inability to move a limb, or inability to understand or formulate speech. Patients who reported commencement in the follow-up period (after the index dates) of new manifestations of a malignancy (beyond that involving skin) were designated to have incident malignancy, and its type was recorded, when known. COPD was defined as those patients who had no evidence of this at baseline, but received this diagnosis in the follow-up period in our out-patient chest clinic, based on obstructive findings in pulmonary function testing, and in whom bronchodilator drugs were initiated.

### 2.6. Data Analysis

Descriptive parameters were shown as mean (±standard deviation [SD]) or in percentages. Owing to skewed distribution, geometric means were used for triglyceride and C-reactive protein values. Two-sided t-tests and Pearson’s chi-square tests were used to analyze the differences between means and proportions of multiple groups and by pairwise comparisons with post hoc Tukey’s HSD tests.

The following cut-off points wereused to define the SUA mid-tertile in non-diabetic men and women (5.2 to <6.4 mg/dL, and 3.9 to <4.91 mg/dL), and in diabetic men and women (4.89 to 6.1 mg/dL, and 4.56 to 5.6 mg/dL), respectively. The development of multiple non-fatal events or death in an individual was counted only as one subject, and nonfatal events identified in patients who subsequently died were not included in analyses of non-fatal events.

A multiple linear regression model was analyzed to seek the best independent covariates of SUA derived from findings of univariate correlation. Logistic regression analyses were used for all-cause mortality and the combined endpoint with associations of sex- and age-adjusted SUA, stratified by gender and presence of diabetes at baseline. Risk estimates (RR) and 95% confidence intervals (CI) were obtained in models that were additionally adjusted for fasting glucose, expressed in terms of 1 − SD increment. A value of *p* < 0.05 on the two-tail test was considered statistically significant. Statistical analyses were performed using SPSS-10 for Windows.

## 3. Results

The study sample consisted of 793 non-diabetic (Table 1A) and 220 diabetic (Table 1B) women and men (age 56.3 ± 9.6, age range 40 to 79 years), free of uncontrolled diabetes or acute and chronic CHD at baseline. Table 1B shows the characteristics of the study sample at baseline examination stratified to gender, SUA tertiles, and presence of type-2 diabetes. In non-diabetic men, the highest SUA tertile had increased BMI, high triglyceride/low HDL-cholesterol ratio, elevated BP, and serum creatinine. Glucose as well as other lipid and non-lipid parameters were similarly distributed across the tertiles. A total of 43 deaths, and nonfatal events in an additional 157 subjects, forming the composite endpoint of 200 events, occurred during a follow-up of 1 to 5.5 (median 3.4) years. Overall, 30 deaths among the non-diabetic sample tended to be lowest in the mid-tertile. In non-diabetic women, the highest SUA tertile was associated with similar alterations, additionally also with elevated fasting glucose, HbA1c, and C-reactive protein, yet disclosing similar HDL-cholesterol values. Moreover, females in the low tertile were (by ~3 years) younger and those in the mid-tertile had shorter body height.

Pearson correlations of SUA with creatinine or total cholesterol within each SUA tertile are shown in Supplementary Table S1. These indicate that circulating creatinine is uniformly highly significantly correlated positively with SUA in both sexes. On the other hand, an absolute lack of correlation between SUA and total cholesterol in the higher two tertiles turned to a positive correlation in the lowest tertile, especially in men (*r* = 0.14, *p* = 0.086).

A multiple linear regression analysis for independent association with baseline SUA was constructed each in of the non-diabetic and diabetic samples comprising creatinine, BMI, fasting glucose, triglycerides, HDL-cholesterol, HbA1c, age, and systolic BP (Table 2). In highly significant models among the non-diabetic sample, the first threestated variables were significantly and positively associated, in addition to triglycerides. Testing for co-linearity among BMI, fasting glucose, and triglycerides yielded negative results in women as well as men. Interaction between these variables was also absent in either sex. In patients with diabetes exhibiting again highly significant regression models, alterations in associations were noted in men regarding conversion to inverse trend in fasting glucose, and attenuated association with triglycerides. In diabetic women, greater disparity was observed compared with non-diabetic women with respect to attenuation of BMI, to assuming significance by age, and the emergence of a significant inverse association between SUA and HDL-cholesterol.

Supplementary Figure S1 depicts the distribution of crude overall mortality (*n* =43) and death rate related to SUA tertiles in the whole sample. Compared with the mid tertile, the lowest tertile revealed an over twofold risk of death, similar to the highest tertile. Table 3 demonstrates the findings in logistic regression analyses for associations of SUA with overall mortality, non-fatal events, and the composite endpoint at follow-up, stratified to gender as well as the diabetic status, independent of age, smoking status, total cholesterol, and presence of hypertension. A significant inverse association with baseline SUA tertile 3 prevailed with respect to nonfatal events in non-diabetic men (RR 0.26) and gender combined (RR 0.40 [95% CI 0.22; 0.72]). In Cox regression analysis for all-cause mortality, when SUA mid-tertile was taken as a reference in the whole sample, the lowest tertile had an RR of 2.01 (95% CI 0.82; 4.93) and, when the highest and lowest tertiles were combined, an RR of 2.24 (95% CI 0.99; 5.08) was obtained, after adjustment of sex, age, smoking status, total cholesterol, and presence of hypertension. The numeric distribution of deaths and nonfatal events is provided in Supplementary Table S2.

### Dichotomized SUA in the Whole Sample as a Predictor of Outcomes

In analyses of non-fatal events in the whole (non-diabetic and diabetic) study sample, using the regression model stated in Table 3, along with diabetes, SUA lowest tertile exhibited a significantly increased risk compared with the uppermost two tertiles combined (RR 1.51 [95% CI 1.02; 2.26]). This association retained significance among non-diabetic or diabetic (RR 3.27 [95% CI 1.07; 9.95]) men, yet was attenuated in women. The combined lower and upper tertiles of SUA tended to predict overall mortality (RR 2.40 [95% CI 0.89; 6.51]) compared with the mid-tertile, additively to significant risks conferred by former smoking and by 1−SD decrement of total cholesterol (RR 1.47 [95% CI 1.07; 1.96]).

**Figure 2:**
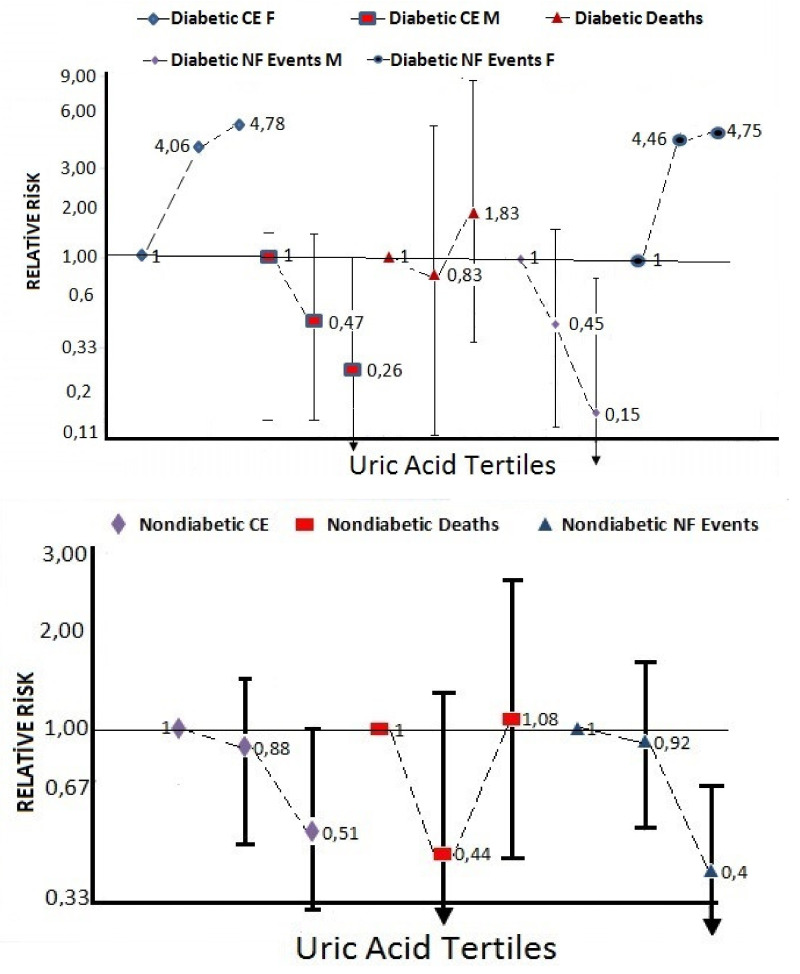
Relative risks (RRs) of serum uric acid tertiles for mortality, non-fatal events, and the composite endpoint in the non-diabetic (lower panel) and the diabetic sample (upper panel). RRs (and 95% CIs) for non-fatal events and the composite endpoint in diabetic patients are depicted separately in the sexes owing to the gender divergence displayed.It can be observed that subsequent non-fatal events and the composite endpoint are inversely related to the baseline uric acid tertiles in the total non-diabetic and the male diabetic sample. Overall mortality carries a U-shaped risk curve regarding uric acid tertiles in the whole sample. Female diabetic patients reveal divergence with respect to non-fatal events and the composite endpoint, showing an, albeit non-significant, tendency to increased risk in the upper two tertiles.

For the composite endpoint in the whole study sample, including diabetes as an independent variable, SUA lowest tertile exhibited a borderline significantly increased risk compared with the two highest tertiles combined (RR 1.38 [95% CI 0.96; 1.98]). This association showed a certain gender disparity among diabetic patients insofar as the low tertile displayed an RR of 2.51 [95% CI 0.86; 7.09]) in men, contrasted to 0.18 [95% CI 0.03; 1.09] in women (Figure 2).

## 4. Discussion

Although the relationship between high uric acid levels and cardiovascular outcomes is known, their relationships with low SUA levelsare not known. We planned to investigate cardiovascular outcomes with low SUA levels. A U-shaped relationship was found between SUA level and all-cause deaths in a study by Hu et al. [12]. Salient findings were that the lowest SUA tertile compared with the higher two tertiles displayed an inverse association for nonfatal cardiac or non-cardiac events and the composite endpoint in middle-aged non-diabetic people or diabetic men, free of baseline CHD. Furthermore, low SUA tertile predicted independently nonfatal events in the entire study sample. The risk of all-cause mortality in non-diabetic subjects tended to exhibit a U-shaped curve. Diabetic women displayed the disparity of a borderline significant positive association of SUA with non-fatal events and the composite endpoint, likely reflecting associated pro-inflammatory state and HDL dysfunction in diabetic status. Exceptions were observed regarding the risk of death, which tended to be conferred by both extreme tertiles, while non-fatal outcomes in diabetic women were predicted by tertiles 2 and 3. Interestingly, the risk for the composite endpoint was significantly increased in women who had discontinued smoking, irrespective of having or not diabetes. Collectively, these findings are consistent with the notion of potential competing outcomes induced by a shared underlying autoimmune activation [9].

### 4.1. Stratified Analysis and Covariates of SUA

With the purpose of eliciting appropriate associations and conclusions, cross-sectional analyses for covariates and longitudinal outcomes had to be made using stratification to diabetes status and gender. Serum creatinine demonstrated the highest correlation and independent association with SUA in each sex, even in each SUA tertile. Although high SUA levels pose a risk for chronic renal failure, the effect of low uric acid levels is unknown. In the study conducted by Mori et al., while a low uric acid level poses a significant risk for chronic kidney failure in women, a high serum uric acid level increases the risk for chronic kidney failure in both sexes [13]. This suggests the concomitant involvement of both SUA and creatinine in the immune complex, for which evidence was previously reported regarding serum creatinine in Turkish women [14]. Hyperfiltrators (generally having low serum creatinine) were characterized by having lower serum total and LDL-cholesterol, uric acid, and left ventricular ejection fraction than persons with normal estimate glomerular filtration rate (eGFR) [15]. Lower SUA is in line with recently reported observations among Turks that uric acid was independently and linearly associated with serum total phospholipids in men and women free of MetS, and that the concentration of phospholipids on HDL predicted the development of MetS [16], thus supporting a notion that a reduced uric acid level may be due to partial inability to assay, secondary to autoimmune activation. Elevated SUA, on the other hand, emerged as a marker of both pro-inflammatory state and HDL dysfunction among Turks [17]. This situation is different from genetic diseases such as xanthinuria with hypouricemia.

In a recent commentary, Puddu and Menotti [18] pointed to the clear curvilinear relationship of creatinine-based eGFR with all-cause mortality rate, evident for people 55 years old or over. They stressed that SUA might be considered for risk predictive purposes to be incorporated into software instruments aimed at indexing and/or following up individuals for primary preventive purposes [19].

### 4.2. Associations Interacted with Diabetic Status and Sex

Our findings generated evidence suggesting that “reduced” SUA levels in non-diabetic people may carry adverse clinical significance. Diabetic status is the major confounder of the presumable underlying autoimmune activation, interacting in women by shifting the weight of adverse events to the higher end of SUA. In agreement with our finding of sex as a second modifier of adverse outcomes, different dose–response relationships of SUA levels with risk of stroke mortality were found for men and women [20]. An interaction between elevated SUA levels and low triglycerides as an independent determinant of systolic BP and modifying the association between SUA and pre-hypertension has recently been reported among Japanese adults [21].

### 4.3. Predictive Value for All-Cause Mortality Risk and Nonfatal Events, as Well as Explanation

SUA proved to be an independent prognostic contributor, both at lower concentrations in non-diabetic people, and at elevated levels, especially in diabetic women. The fact that lower SUA concentrations did not mediate future events because of a reduced anti-oxidant capacity can be argued based on our analysis showing significant associations with fasting glycemia, elevated triglycerides, and BMI, all mediators of oxidant stress. Therefore, reduced SUA levels are due presumably to partial assay inability mediated autoimmune activation rather than representing a lowered anti-oxidant effect. The interpretation that SUA may act as a protective factor against the development of incident osteoporotic fractures (in Korean men) because of its anti-oxidant properties [22] seems misplaced, and rather reflects the absence of autoimmune processes at high SUA levels.

Our hypothesis is further supported by the observation that total cholesterol was significantly inversely associated with mortality in non-diabetic individuals. This is in agreement with the reported precipitous decline in serum levels of total and LDL cholesterol preceding the clinical rheumatoid arthritis incidence and the association of this development with higher cardiovascular risk [23], as well as with the observation in the TARF [24] that Lp(a) was significantly reduced in the period preceding new-onset diabetes and with HOMA index. This is in line also with the report within the ARBITER 6-HALTS trial on the progression of carotid intima-media thickness with greater LDL-cholesterol reduction during statin and ezetimibe therapy [25] in a patient group characterized by susceptibility to impaired glucose tolerance.

Evidence indicates that several polypeptides/proteins that induce autoimmune responses, including serum Lp(a) [26], creatinine [14], HbA1c [27], and thyroid stimulating hormone [28], were associated with subsequent cardio-renal and metabolic risk [9].

### 4.4. Former Smoking Status, a Clear Contributor to Adverse Outcomes

A side finding of clinical interest was the strong independent association of former smoking status with future outcomes in non-diabetic and, more so, diabetic women, further supporting the autoimmune basis of the outcomes and the potential inhibition of this process by active smoking [29]. Reports paralleling the current finding are available regarding Polish women with respect to MetS [30] and Turkish adults regarding type-2 diabetes and MetS [31]. Worthy of note is that, though non-diabetic male smokers compared with never smokers presented an excess risk for the composite endpoint, former male smokers were confronted with roughly 40% greater risk than current smokers, consistent with a view of inhibition by active smoking of autoimmune processes [29] in non-diabetic males as well.

### 4.5. Conceivability of Various Chronic Diseases Partially Rising from Autoimmune Activation

We have investigated whether or not and how possibly related outcomes as fatal and non-fatal cardiac and diverse non-cardiac (COPD, malignancies) events were associated with SUA in this study. Such an attempt may not be far-fetched because it was concluded in a recent meta-analysis that MetS is associated with increased risk of common cancers [32], an association of MetS with colorectal neoplasm was confirmed in a second meta-analysis [33], and the fact that patients with heart failure have an increased risk of incident cancer [34]. It is also recognized that one-third of patients with COPD are estimated to be never smokers, but are thought to be exposed to biomass fumes, air pollutants, and so on. Beyond this, an enhanced pro-inflammatory state associated with obesity might also result in COPD among non-smokers, a tendency reported among Korean men [35].

### 4.6. Implications

Excess risk of non-fatal events and mortality associated with “reduced” SUA concentrations in middle-aged people should urge inquiry into the possible existence of an underlying autoimmune response, which requires at least the identification and elimination of the usage of drugs, such as antipsychotics and antidepressants [36], as well as discovering newer immunoassay methods and preventive measures.

### 4.7. Limitations and Strength

Admittedly, the number of events and deaths was relatively limited; informative results could nonetheless be obtained, shedding light on controversies prevailing on the prognostic significance of SUA levels. The source of data being a single-centre and insufficient follow-up time are further limitations. Though consideration of the combination of heterogeneous outcomes may seem a limitation, utilizing the combination has a rationale. Using a single SUA measurement without taking into account within-person variability may have introduced some regression dilution bias, leading though to an underestimated risk. The applicability of the current findings to population subsets not prone to impaired glucose tolerance remains to be discerned. Analysis by the exclusion of CHD patients at baseline and stratification to both diabetic status and gender are major strengths that allowed the emergence of meaningful results. Demonstration of the predictive value of low SUA levels for outcome risk, independent of traditional risk factors, is a further strength.

## 5. Conclusions

Longitudinal analysis of middle-aged hospital attendees without or with type-2 diabetes for overall mortality and a composite endpoint of death, non-fatal cardiac, or non-cardiac disease risk documented in combined gender that “reduced” SUA assays contributed significantly to non-fatal outcomes (RR around 1.5), and at borderline significance to the composite endpoint, additively to conventional cardiovascular risk factors. This association may be attributable to the underlying phenomenon of autoimmune activation.

## Figures and Tables

**Table 1:** (**A**) Characteristics of the study sample (*n* = 1013), stratified to serum uric acid tertiles, gender, and non-diabetic group at baseline. (**B**) Characteristics of the study sample (*n* = 1013), stratified to serum uric acid tertiles, gender, and presence of diabetes at baseline.

(A)								
	Men (*n* = 453)				Women (*n* = 560)			
	Low Tertile1	Mid Tertile2	High Tertile3			Low Tertile1	Mid Tertile2	High Tertile3
	111 (*n*) (4.49 mg/dL)	120 (*n*) (5.76 mg/dL)	118 (*n*) (7.29 mg/dL)	*p-*Value		165 (*n*) (3.34 mg/dL)	154 (*n*) (4.54 mg/dL)	125 (*n*) (6.08 mg/dL)
	Mean ± SD	Mean ± SD	Mean ± SD	*Men*	*Women*	Mean ± SD	Mean ± SD	Mean ± SD
*Non-Diabetic Sample*	*n* = 349					*n* = 444		
Age, years	56.4 ± 10.3	54 ± 9.5	55.7 ± 9	0.070	0.002	54.4 ± 10	56.7 ± 9	58.3 ± 8.7
Body mass index (kg/m^2^)	26.8 ± 3	26.9 ± 3	28.1 ± 3,7	0.008	<0.001	26.1 ± 4	28 ± 5	28.2 ± 4
Systolic BP, mmHg	126 ± 14	128 ± 12	131 ± 13	0.027	0.032	125 ± 17	127 ± 17	130 ± 16
Diastolic BP, mmHg	78 ± 8.4	79 ± 8	81 ± 9	0.030	0.032	76.4 ± 10.5	78 ± 10.5	80 ± 10.5
Total cholesterol, mg/dl	209 ± 39	216 ± 44	217 ± 45	0.330	0.480	218 ± 43	224 ± 53	225 ± 49
HDL-cholesterol, mg/dl	48 ± 12	48 ± 12	45 ± 10	0.008	0.051	59 ± 14	57 ± 13	55 ± 16
Fast triglycerides, mg/dl	152.3 ± 92.3	164.1 ± 83.7	184.8 ± 103.8	0.030	0.009	132.2 ± 78.6	152.8 ± 82.9	162.5 ± 98.1
LDL-cholesterol, mg/dl	125 ± 36	127 ± 37	139 ± 40	0.160	0.670	135 ± 35	138 ± 41	139 ± 40
HbA1c, %	5.45 ± 0.34	5.5 ± 0.4	5.52 ± 0.4	0.320	<0.001	5.41 ± 0.4	5.46 ± 0.4	5.59 ± 0.4
Fasting glucose, mg/dl	96.5 ± 11	97.3 ± 9	98.2 ± 11.4	0.120	<0.001	93 ± 10	98 ± 10	100 ± 10
Creatinine, mg/dl	0.96 ± 0.18	1.05 ± 0.18	1.09 ± 0.15	<0.001	<0.001	0.78 ± 0.16	0.824 ± 0.17	0.88 ± 0.16
C-reactive protein, mg/L	2.86 ± 2.9	3.92 ± 10.1	4.02 ± 4.79	0.538	0.030	2.41 ± 2.46	3.48 ± 4.67	3.85 ± 3.49
Current smoking, *n* (%)	38 (34.2%)	35 (29.1%)	34 (28.8%)	0.300	0.990	27 (16.4%)	24 (15.6%)	21 (16.8%)
LVEF, percent (%)	62 ± 7	62 ± 8	61 ± 8	0.670	0.050	62 ± 6	62.5 ± 5	60.7 ± 9
Overall mortality, *n* (%)	7 (6.3%)	2 (1.7%)	9 (7.6%)	0.073	0.820	5 (3%)	3 (2%)	3 (2.4%)
Composite endpoint, *n* (%)	25 (22.5%)	17 (14.2%)	16 (13.6%)	0.140	0.590	26 (15.8%)	28 (18.2%)	19 (15.2%)
**(B)**								
	**42 (*n*)** **(4.15 mg/dl)**	**34 (*n*)** **(5.77 mg/dL)**	**28 (*n*)** **(7.43 mg/dL)**	***p-*Value**		**21 (*n*)** **(3.47 mg/dL)**	**40 (*n*)** **(4.61 mg/dL)**	**55 (*n*)** **(6.41 mg/dL)**
	**Mean ± SD**	**Mean ± SD**	**Mean ± SD**	***Men***	***Women***	**Mean ± SD**	**Mean ± SD**	**Mean ± SD**
***Diabetic Sample***	***n* = 104**					***n* = 116**		
Age, years	57.3 ± 9	53.9 ± 8	58.2 ± 10.4	0.150	0.008	58.4 ± 8	56.8 ± 8.8	62.4 ± 8.9
Body mass index, kg/m^2^	28 ± 4	28 ± 3	28.8 ± 3,6	0.510	0.390	28.5 ± 3.5	30.3 ± 4.5	29.6 ± 4.8
Systolic BP, mmHg	133 ± 15	128 ± 11	136.5 ± 12.6	0.062	0.340	128 ± 18	129 ± 17	134 ± 21
Diastolic BP, mmHg	83 ± 11	80 ± 9	85 ± 10.7	0.130	0.200	77 ± 13	80.6 ± 8.4	82 ± 9
Total cholesterol, mg/dL	212 ± 52	220 ± 104	220 ± 51	0.880	0.650	215 ± 35	225 ± 49	217 ± 46
HDL-cholesterol, mg/dL	46.5 ± 12.5	44 ± 9.5	47.5 ± 11	0.510	0.410	53.4 ± 14.5	53.6 ± 11.5	50.5 ± 11
Fast triglycerides, mg/dL	188.7 ± 114.1	238.1 ± 259.6	180.9 ± 70.2	0.334	0.910	169.7 ± 90.6	180.6 ± 102.5	176.1 ± 86.9
LDL-cholesterol, mg/dL	125 ± 46	121 ± 28	134 ± 39	0.430	0.970	130.6 ± 34	132.5 ± 41	133 ± 40
HbA1c, %	6.97 ± 1.0	6.82 ± 0.8	6.41 ± 0.7	0.030	0.084	6.94 ± 0.83	6.39 ± 0.74	6.60 ± 0.98
Fasting glucose, mg/dL	151 ± 46	136 ± 36	123 ± 25	0.010	0.074	159 ± 58	131 ± 34	140 ± 46
Creatinine, mg/dL	0.89 ± 0.20	1.03 ± 0.15	1.10 ± 0.17	<0.001	0.079	0.75 ± 0.15	0.79 ± 0.18	0.85 ± 0.21
C-reactive protein, mg/L	3.81 ± 3.57	2.83 ± 3.76	4.91 ± 5.73	0.343	0.106	2.69 ± 1.51	4.48 ± 3.50	6.76 ± 8.40
Current smoking, *n* (%)	9 (24%)	8 (24%)	7 (27%)	0.160	0.540	6 (28.6%)	7 (18%)	14 (26%)
LVEF, percent (%)	59 ± 10	62 ± 3	61 ± 6.6	0.300	0.098	62 ± 3.3	61.5 ± 7	58 ± 8
Overall mortality, *n* (%)	3 (7%)	1 (3%)	2 (7.7%)	0.690	0.400	1 (4.8%)	1 (2.6%)	5 (9.3%)
Composite endpoint, *n* (%)	**16 (41%)**	6 (18%)	4 (15%)	0.032	0.220	3 (14.3%)	11 (33.3%)	18 (33.3%)

Numbers in boldface denote significant difference from the other two tertiles. BP = blood pressure, LVEF = left ventricular ejection fractions. Uric acid normal ranges: 3.5–7.2 mg/dl.

**Table 2:** Linear regression analysis for independent associations with baseline serum uric acid (SUA) (*n* =927), stratified by diabetic status and gender.

	Men, *n* = 319 *			Women, *n* = 408 *		
***Non-Diabetic Subjects***	ß coeff.	SE	*p*-Value	ß coeff.	SE	*p*-Value
Creatinine, 0.20 mg/dL	**0.43**	0.08	<0.001	**0.43**	0.07	<0.001
Body mass index,4 kg/m^2^	**0.20**	0.09	0.018	**0.12**	0.06	0.026
Fasted glucose, 25 mg/dL	**0.40**	0.17	0.028	**0.60**	0.16	0.002
Fasted triglycerides, 30 mg/dL	*0.94*	0.84	0.052	**0.91**	0.82	0.041
HDL-cholesterol, 12 mg/dL	−0.04	0.08	0.640	−0.07	0.06	0.210
HbA1c, 0.8 %	0.04	0.16	0.820	*0.24*	0.14	0.070
Age, 11 years	−0.13	0.09	0.120	0.11	0.08	0.130
Systolic BP, 25 mmHg	0.15	0.13	0.270	0.25	0.1	0.710
Constant	−0.93	1.68	0.580	**−3.35**	1.22	0.006
r^2^	15%, *p* < 0.001			19%, *p* < 0.001		
***Diabetic sample***	*n* = 95			*n* = 105		
Creatinine, 0.20 mg/dl	**0.60**	0.14	<0.001	*0.27*	0.14	0.061
Body mass index,4 kg/m^2^	*0.28*	0.15	0.084	0.07	0.11	0.530
Fasted glucose, 25 mg/dl	−0.16	0.10	0.110	−0.08	0.10	0.470
Fasted triglycerides, 30 mg/dL	*−0.60*	0.93	0.950	0.43	0.93	0.990
HDL-cholesterol, 12 mg/dL	−0.06	0.18	0.670	**−0.40**	0.13	0.003
HbA1c, 0.8%	−0.15	0.14	0.310	−0.06	0.13	0.670
Age, 11 years	0.33	0.16	0.860	**0.43**	0.15	0.008
Systolic BP, 25 mmHg	0.10	0.25	0.670	0.05	0.18	0.770
Constant	2.64	2.7	0.330	3.51	2.26	0.120
r^2^	28%, *p* < 0.001			18%, *p* = 0.002		

* Missing values in 5.6% of the sample. Numbers in boldface denote significantly and those in italics borderline significantly associated values.

**Table 3:** Logistic regression analysis of serum uric acid tertiles for outcomes, by diabetic status and gender (*n* =1013). RR, relative risk; CI, confidence interval.

	Non-Diabetic						Diabetic Subjects					
	Total *n* = 793		Men *n* = 349		Women *n* = 444		Total *n* = 220		Men *n* = 104		Women *n* = 116	
	RR	95% CI	RR	95% CI	RR	95% CI	RR	95% CI	RR	95% CI	RR	95% CI
*Composite endpoint* (*n*)	138		64		74		62		28		34	
Sex, male	1.11	0.71–1.72					1.76	0.85–3.66				
Age, 10 years	**2.10**	1.66–2.67	**2.32**	1.58–3.36	**1.89**	1.38–2.62	**1.63**	1.10–2.43	*1.74*	0.98; 3.11	1.32	0.72–2.32
Uric acid tertile 2	0.88	0.45–1.43	0.86	0.41–1.84	0.94	0.50–1.79	0.99	0.36–2.02	0.47	0.15; 1.52	4.06	0.66–24.9
Uric acid tertile 3	**0.51**	0.31–0.86	*0.47*	0.22–1.01	0.57	0.28–1.17	0.85	0.37–2.19	*0.26*	0.06; 1.11	*4.78*	0.08–28.5
Current vs. never smoking	**1.83**	1.06–3.15	**2.44**	1.14–5.21	1.36	0.59–3.13	*2.04*	0.90–4.66	**7.62**	1.90; 30.6	0.67	0.19–2.32
Former vs. never smoking	**3.32**	1.81–6.09	**3.58**	1.57–8.19	**3.57**	1.32–9.66	**7.11**	2.91–17.4	**8.37**	2.26; 31.0	**16.0**	2.41–106
Hypertension, yes/no	**3.00**	1.94–4.65	**2.23**	1.17–4.27	**3.75**	2.04–6.88	*1.88*	0.93–3.78	1.03	0.35; 3.08	**3.31**	1.19–9.25
Total cholesterol, 35 mg/dL	0.93	0.78–1.07	0.78	0.61–1.01	1.00	0.81–1.19	0.93	0.78–1.11	1.04	0.84; 1.28	0.78	0.53–1.15
*All-cause mortality (n)*	30		19		11		13		6		7	
Sex, male	0.76	0.33–1.75					1.23	0.29–5.19				
Age,10 years	**1.69**	1.07–2.67	**1.99**	1.04–3.81	1.33	0.67–2.62	**1.95**	1.06–4.23	2.43	0.63–9.39	1.61	0.51–5.10
Uric acid tertile 2	0.44	0.15–1.31	0.41	0.19–11.1	0.50	0.11–2.28	0.83	0.13–5.36	0.61	0.03–11.1	0.57	0.03–11.2
Uric acid tertile 3	1.08	0.46–2.53	1.52	0.52–4.47	0.62	0.14–2.75	1.83	0.36–9.27	2.97	0.17–51.4	2.01	0.19–21.2
Current vs. never smoking	*2.79*	0.99–7.81	**8.23**	1.55–43.8	0.74	0.61–3.69	1.46	0.24–9.02	NS	Too few	prot.	Too few
Former vs. never smoking	**4.84**	1.76–13.3	**9.77**	1.81–52.7	3.08	0.56–17.1	*4.36*	0.95–20.0	NS	Too few	3.59	0.54–24.1
Hypertension, yes/no	1.23	0.54–2.81	0.87	0.30–2.57	1.84	0.48–7.08	0.39	0.11–1.42	0.17	0.01–7.17	0.92	0.17–4.87
Total cholesterol, 35 mg/dL	**0.70**	0.51–0.93	**0.61**	0.38–0.99	0.75	0.49–1.15	0.68	0.41–1.15	0.21	0.07–1.37	1.04	0.55–2.00
Non-fatal events * (n)	108		45		63		49		22		27	
Age,10 years	**2.16**	1.66–2.81	**2.26**	1.48–3.46	**2.06**	1.45–2.92	*1.49*	0.97–2.30	1.63	0.87–3.05	1.12	0.59–2.12
Uric acid tertile 2	0.92	0.55–1.55	0.88	0.39–1.98	0.99	0.50–1.97	0.93	0.37–2.33	0.45	0.14–1.65	4.46	0.57–34.7
Uric acid tertile 3	**0.40**	0.22–0.72	**0.26**	0.10–0.69	0.55	0.25–1.18	0.66	0.26–1.71	**0.15**	0.03–0.84	4.75	0.61–36.7
Current vs. never smoking	1.63	0.88–3.00	1.73	0.73–4.10	1.50	0.61–3.69	2.05	0.85–4.94	**5.99**	1.40–25.6	0.78	0.22–2.84
Former vs. never smoking	**2.78**	1.41–5.47	*2.51*	0.99–6.37	**3.70**	1.29–10.8	**6.70**	2.57–17.5	**6.12**	1.57–23.8	**18.1**	2.53–130
Hypertension, yes/no	**3.65**	2.22–5.99	**3.03**	1.42–6.48	**4.25**	2.19–8.28	**2.68**	1.19–6.03	1.59	0.47–5.31	**5.47**	1.55–19.3
Total cholesterol, 35 mgdL	1.00	0.84–1.15	0.90	0.66–1.19	1.04	0.84–1.28	0.97	0.81–1.15	1.07	0.87–1.37	0.73	0.48–1.11

* Sex was not significantly associated. Current smoker; already smoking; former smoker; quit smoking; never smoking; never smoked. Numbers in boldface denote significantly and those in italics borderline significantly associated values.

## Data Availability

The datasets generated for this study are available on request to the corresponding author.

## Supplementary Materials

The following are available online at https://www.mdpi.com/article/10.3390/medicina57040361/s1.
