# Supplementary material for: Low Serum Uric Acid Predicts Risk of a Composite Disease Endpoint
Source: Medicina (Kaunas). 2021 Apr 8;57(4):361. doi: 10.3390/medicina57040361 (PMC8068308; doi:10.3390/medicina57040361)
Supplement: Supplementary file 1 [file medicina-57-00361-s001.pdf]

Suppl. Table S1. Pearson correlations between SUA tertiles (n=1013) and creatinine or total cholesterol

		**Tertile 1** (n=339)		**Tertile 2** (n=348)		**Tertile 3** (n=326)	
		r	p-value	*r*	p-value	*r*	p-value
Creatinine	Total	**0.40**	<0.001	**0.51**	<0.001	**0.44**	<0.001
	Male	**0.19**	0.02	0.04	0.59	*0.16*	0.056
	Female	**0.17**	0.02	0.09	0.20	**0.27**	<0.001
Total cholesterol	Total	0.03	0.65	-0.05	0.37	-0.04	0.45
	Male	*0.14*	0.086	-0.03	0.74	0.02	0.86
	Female	0.08	0.29	0.05	0.49	-0.04	0.63

Suppl. Table S2. Distribution of deaths and nonfatal events in the study sample

	**Deaths**	**Non-fatal events**			
		**Total**	Male/Female	**Non-diabetic**	**Diabetic**
Myocardial infarction	9	28	20/8	16	12
Coronary heart disease	0	90	33/57	63	27
Stroke	5	7	4/3	6	1
Heart failure	4	18	4/*14*	12	6
Pulmonary embolism	1	0	0/0	0	0
Chronic obstructive pulmonary disease	5	8	2/6	5	3
Malignancy	10	6	4/2	6	0
Others or indeterminate	9	0	0/0	0	0
total	43	157	67/90	108	49

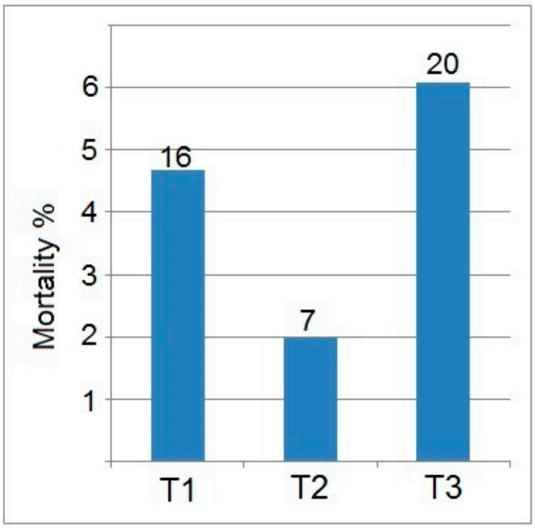

Supplementary Figure S1. Diagram depicts the crude all-cause mortality for tertiles of serum uric acid in the whole study sample (n=1013). Values above the columns indicate the number of recorded deaths. Compared to the mid tertile, the lowest tertile (<5.0 and <4.0 mg/dl in men and women) displayed excess risk of death, similar to the highest tertile (p-value 0.026).
